# Systematic Review of Leiomyomas of the Upper Extremity: Evaluating the Role of Ultrasound in Preoperative Diagnosis

**DOI:** 10.1177/15589447261433068

**Published:** 2026-04-07

**Authors:** Kenzie J. MacNeil, Todd W. J. Dow, Katie B. Ross, Donald H. Lalonde

**Affiliations:** 1 Faculty of Medicine, Dalhousie University, Halifax, NS, Canada; 2 Division of Plastic and Reconstructive Surgery, Dalhousie University, Halifax, NS, Canada; 3 Division of Plastic and Reconstructive Surgery, Dalhousie University, Saint John, NB, Canada

**Keywords:** hand, anatomy, ultrasound, leiomyoma, soft tissue mass, WALANT

## Abstract

Leiomyomas are rare tumors that can occur in various locations, including the upper extremity. Their resemblance to other soft tissue masses can complicate diagnosis, making preoperative imaging essential. This systematic review and case review from our institution evaluates the diagnosis and treatment of upper extremity leiomyomas, with a focus on ultrasound as an underused but valuable diagnostic tool. A comprehensive search of MEDLINE, Embase, PubMed, and The Cochrane Library was conducted from inception to February 1, 2025. Data extracted included presenting symptoms, lesion location, diagnostic tools, histopathology, treatments, and patient outcomes. A total of 1268 articles were reviewed, yielding 105 manuscripts covering 212 cases of upper extremity leiomyomas. The mean age was 48.4 years, with a male predominance (60.7%). Leiomyomas were symptomatic for an average of 58.1 months prior to intervention. The most common locations were the hand and fingers (62.9%). Ganglion cysts were the most frequent misdiagnosis (25.2%). Among the 154 studies describing diagnostic imaging, only 17 (11.0%) used ultrasound while magnetic resonance imaging (MRI; n = 54, 35.1%) was the most commonly used. Surgical excision was the primary treatment, with no reported recurrences. The mean follow-up was 27.3 months. Although MRI remains the gold standard diagnostic tool for upper extremity leiomyomas, ultrasound offers a noninvasive, cost-effective, and accessible alternative. Expanding the use of ultrasound in resource-limited settings may improve early diagnosis, streamline management, and enhance outcomes. Our case demonstrates the usefulness of ultrasound examination for diagnosis and management.

## Introduction

Leiomyomas are benign tumors originating from smooth muscle, and although they are most commonly found in the uterus, they can also develop in any tissue containing smooth muscle, including the extremities.^1^ Leiomyomas of the hand are exceptionally rare and often pose a diagnostic challenge.^2,3^ Although nonvascular leiomyomas tend to develop from smooth muscle in the skin or subcutaneous tissues, vascular leiomyomas (angioleiomyomas) are more likely to arise from the walls of blood vessels, most frequently veins.^4-7^ These tumors are typically slow-growing, solitary, and benign. The vascular variant of leiomyoma may produce additional symptoms including pain, and in some cases, can compress adjacent nerves leading to neurologic symptoms similar to carpal tunnel syndrome (CTS) or Guyon canal syndrome.^8,9^

The rarity of angioleiomyomas and leiomyomas in the upper extremity, combined with their nonspecific presentation, underscores the importance of a high level of clinical suspicion when evaluating unexplained hand masses. Given the variety of conditions that can mimic these tumors, the differential diagnosis is broad and includes both benign and malignant entities.^3^ The intricate anatomy and functional significance of the hand make early recognition and prompt management critical to prevent long-term complications such as restricted range of motion, nerve compression, or deformity.^10^

Imaging techniques such as magnetic resonance imaging (MRI) and high-frequency ultrasound (US) have become increasingly useful in identifying soft tissue tumors and distinguishing them from other pathologies.^11^ Although MRI is often preferred for evaluating deeper soft tissue masses, its limited availability in resource-constrained settings can hinder timely diagnosis and treatment. Ultrasound has gained popularity for its ability to provide real-time imaging and assess vascular involvement, making it particularly valuable in cases of suspected angioleiomyoma.^9,12^ Despite advances in US imaging and its increasing adoption, clinicians remain somewhat hesitant to rely on it as a first-line diagnostic in hand clinics, in part due to limited operator expertise, perceived complexity, capital expenditure and traditional reliance on radiology.^13^

The aim of this article is to provide a systematic review of published cases of leiomyoma and angioleiomyoma of the upper extremity, exploring the clinical presentation, diagnostic tools and management strategies for these soft tissue lesions. This article will provide a special focus on the use of US for initial diagnosis and present video of our own use of US in the treatment of a leiomyoma of the thumb. Through this review, we will provide increased awareness of this rare condition and improve the clinical approach to hand tumors in resource-limited environments. We hypothesized that the use of US for the diagnosis of upper extremity leiomyomas has been historically underreported and underused, despite its potential accuracy and accessibility compared to MRI.

## Methods

### Protocol and Eligibility Criteria

This systematic review was conducted in accordance with the Preferred Reporting Items for Systematic Reviews and Meta-Analysis (PRISMA) guidelines.^14^ The population consisted of human patients with histopathologically confirmed leiomyomas of the upper extremity. There were no restrictions regarding age, country of publication, or language.

### Search Methodology and Study Selection

A systematic literature search of PubMed, MEDLINE, Embase, and Cochrane Register electronic databases was conducted using keywords and Medical Subject Headings (MeSH) terms for “leiomyoma,” or “angioleiomyoma” AND “upper extremity,” with the assistance of a medical librarian. Articles published from inception to February 1, 2025, were included, and all references of these were screened for inclusion. Two authors (K.M. and T.D.) independently completed the initial screening and full-text review, with a third author (D.L.) resolving any conflicts.

### Data Extraction

Data extraction was performed by two authors (K.M. and T.D.) using a Microsoft Excel spreadsheet. The data extracted included variables related to study characteristics (author, year, country, journal, and language of publication, and study design) and population (age, sex, lesion location, hand dominance, symptoms, duration of symptoms, investigations, histopathological results, treatment, follow-up, and outcomes).

### Statistical Analysis

When available, categorical variables were summarized as counts and percentages, while continuous variables were presented as median and interquartile ranges for nonnormally distributed variables or as mean values and standard deviations (SDs) for normally distributed variables.

### Case Presentation

Written consent was obtained from the patient for both case presentation and media use.

## Results

**Figure 1.**
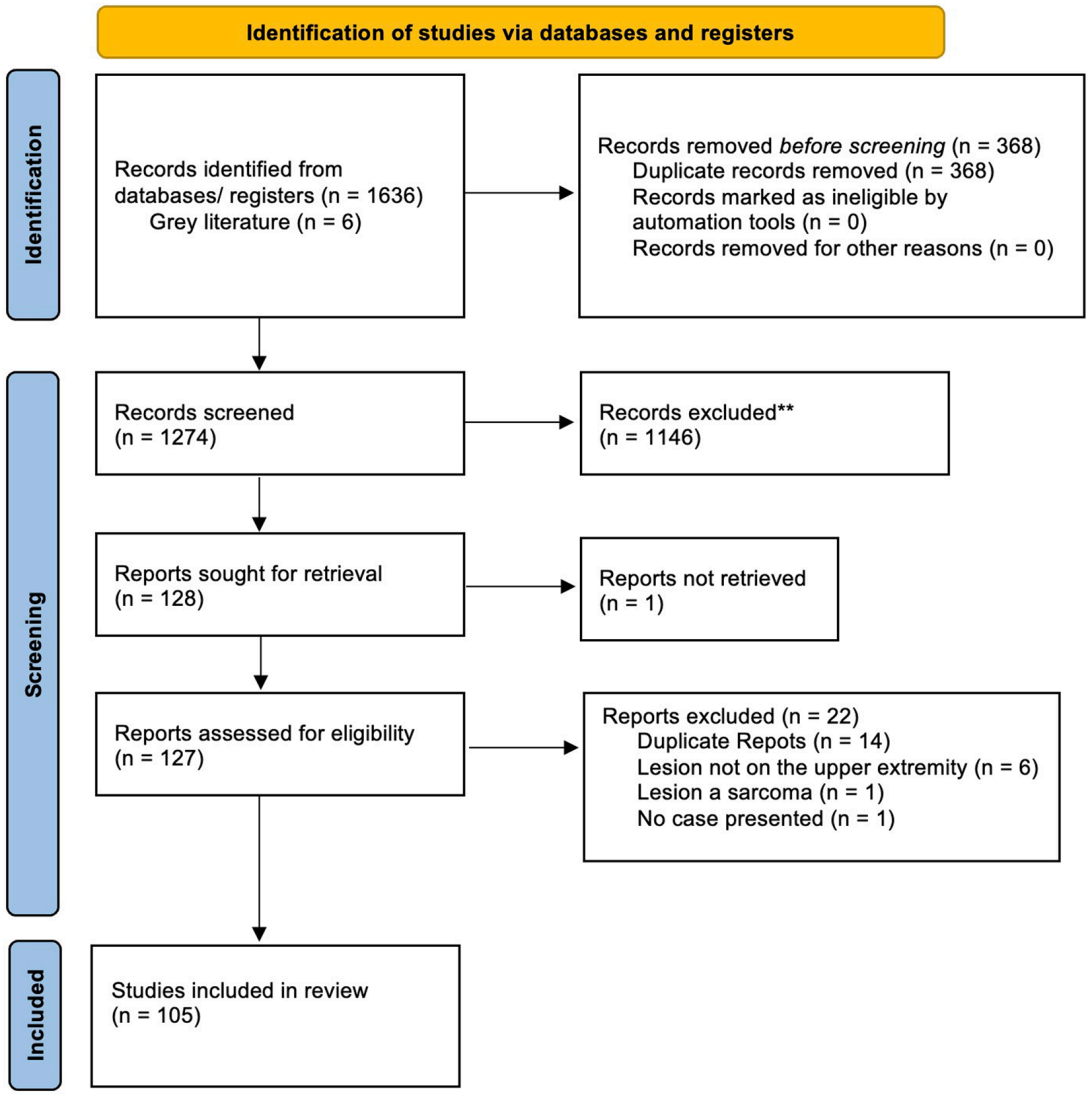
Flowchart of study inclusion using Preferred Reporting Items for Systematic Reviews and Meta-Analyses (PRISMA) guidelines.

A total of 1642 articles were identified in our initial search, of which 105 met the inclusion criteria (Figure 1). Articles were excluded if they did not present cases of leiomyomas in the upper extremity or if they presented a population of patients with leiomyomas without distinguishing those with lesions in the upper extremity. References for the included articles can be found in Supplementary Material. The articles were published between 1954 and 2024. The vast majority of published articles were single case reports (n = 87, 82.9%). Cases were presented from 26 countries, with the United States (n = 31, 29.5%) being the most common (Supplementary Table 1). In the cases presented within retrospective review articles, data were often provided as means for age and frequencies for location and sex. These were used in the cumulative analysis, even if specific details could not be accurately assigned to individual patients.

Within the 105 articles, 212 unique cases were identified (Supplementary Table 1). With the addition of our case, this provided a population of 213 patients. The recorded location of these lesions was as follows: 40% occurred in the digits, 25% in the palm or thenar/hypothenar regions, 15% at the wrist, and 10% in the forearm or arm, with approximately 70% located on the volar surface; the remaining cases did not have a specific location reported. The mean age was 48.4 ± 16.5 years (5-86 years), and males were more commonly affected (60.7%). Lesions were symptomatic for an average of 58.1 ± 65.7 months (range: 0.5-420 months) prior to intervention. The majority (54.2%) of patients presented with pain, while the second most common symptom was swelling (26.8%). A history of trauma was reported by only 6 patients and was considered not to be a contributing factor. The most common misdiagnosis at presentation was a ganglion cyst (25.2%). Misdiagnosis rates were calculated by comparing the initial clinical diagnosis to the final histopathologic diagnosis, which served as the gold standard. Hand dominance was reported in 35 (16.5%) of cases and did not correlate with the side of the lesion.

Of the 213 lesions, 115 cases (54.0%) specified the side on which the lesion occurred. There was no apparent lateralization, as lesions were evenly distributed with 58 cases on the right hand and 57 cases on the left. When categorizing the upper extremity into “upper arm,” “elbow,” “forearm,” “wrist,” “hand” and “digits,” the hand was the most common site for a lesion (n = 87, 46.3%), followed by the digits (n = 69, 32.4%). Within the hand, the palm was approximately 4 times more common than the dorsum. Among the digits, the index finger was the most commonly involved (33.3%).

Diagnostic tools used to investigate the lesions varied considerably among the reviewed studies. Only 15 (7.0%) of the studies reported performing a biopsy. Of the 154 studies that described their diagnostic imaging, only 17 (11.0%) used US, with MRI (n = 54, 35.1%) being the most commonly used diagnostic tool. Among the 17 articles that described the use of US, the majority of cases used this imaging modality to assess Doppler flow of the lesion. Calcifications were detected by imaging or final histopathologic analysis in 13 lesions (6.1%). The use of MRI and US as investigation modalities began with cases published in 1997 or later. Of these 127 cases, 30 did not report any form of imaging investigations, 54 (42.5%) used MRI and 17 (13.4%) used US. There was no increasing utilization in published literature between 1997 and the most recently reviewed manuscript. Surgical excision was the primary treatment, with no reported recurrences. The mean follow-up was 27.3 ± 24.9 months (range: 0.25-84 months).

## Case Presentation

A 56-year-old male presented with a 4- to 5-year history of a slow, progressively enlarging growth on his left thumb. He described the lesion as nontender, soft, and somewhat compressible.

On examination, the lesion was located on the ulnar aspect of his left thumb, overlying the proximal phalanx. It did not limit his range of motion for extension or flexion of the metacarpophalangeal joint or the interphalangeal joint. However, he described difficulty with power-grip, as the lesion would become compressed and partially hinder his work as a mechanic. There were no changes to the overlying skin; his thumb exhibited normal capillary refill, turgor, and warmth. Transillumination was attempted, but the lesion did not transilluminate. He had a negative Tinel sign and did not report any sensory deficits in the thumb. Interestingly, the patient described that when he worked outside in the cold Canadian winter, he noticed the lesion would shrink and, on occasion, disappear completely. Upon returning to warmth, he observed that the lesion would revert to its original size.

A bedside US examination was performed (Video 1). The US demonstrated a well-encapsulated lesion in the subcutaneous tissue overlying the ulnar aspect of the proximal phalanx of the left thumb. The US findings ruled out a ganglion cyst, as the lesion was not anechoic (fluid-filled dark area) or homogeneous. The lesion also was clearly separate from underlying tendons and bone.

**Figure 2.**
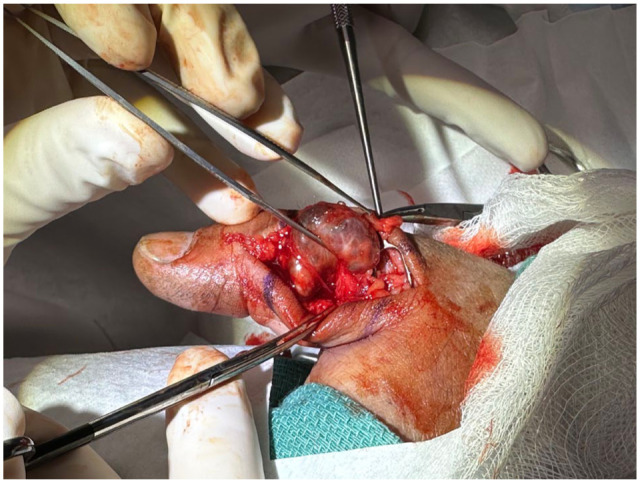
A 56-year-old male with an angioleiomyoma of the left thumb.

Wide-Awake Local Anesthesia No Tourniquet (WALANT) surgery was performed to excise the lesion in the clinic following the US examination. Preoperative US demonstrated the lesion was adjacent to, but not involving, the digital artery, confirming safe excision under WALANT conditions. In cases with suspected arterial encasement or compromised flow, microvascular reconstruction may be required. Video 2 demonstrates the expert application of local anesthesia for WALANT surgery by Dr Don Lalonde (Video 2). Surgical excision revealed a well-encapsulated, gray, vascular lesion involving an ulnar digital vein of the left thumb (Figure 2). The vein was mobilized both proximally and distally and ligated, allowing for complete resection of the lesion. The patient was discharged home shortly after the surgery with a simple bandage.

Histopathology demonstrated an irregular portion of gray tissue measuring 2.2 × 1.6 × 1.5 cm. The specimen revealed medium-sized blood vessels with thrombosis, intermixed with benign spindle cells and a rim of mature adipose tissue. CD31 staining highlighted the vasculature, myosin staining highlighted the spindle cells, and HMB45 positivity in all elements suggested an angioleiomyoma. The patient recovered well from the surgery and returned to work in 1 week. At the 14-month follow-up, no recurrence has been noted.

## Discussion

Leiomyomas of the upper extremity, particularly in the hand and digits, are rare but clinically significant entities.^15^ These tumors, which arise from smooth muscle tissue, are most commonly seen in the lower extremities or the uterus.^1^ The rarity of cases in the upper extremity has made it challenging to compile comprehensive data on their clinical behavior, management, and prognosis. Our systematic review of 213 cases provides important insights into the global clinical experience with leiomyomas in the upper extremity, enhancing the understanding of their presentation and management.

Our findings support the established knowledge that leiomyomas in the upper extremity commonly present with pain and swelling.^16^ In the cases reviewed, pain was the most frequently reported symptom (54.2%), and It is believed that cutaneous leiomyomas are painful because contraction of the smooth muscle within the tumor compresses nearby cutaneous nerves, producing localized or radiating pain often triggered by cold or touch^17-19^ The occurrence of swelling as the second most common symptom (26.8%) also underscores the progressive growth of these tumors. Interestingly, trauma was infrequently reported as a contributing factor (6 patients), suggesting that, contrary to some hypotheses, repetitive trauma or injury may not play a significant role in the development of these tumors. Some authors have speculated that repeated microtrauma may contribute to tumor development. Weisman referred to a study by Bickel on soft tissue tumors in the hand and found 7 painful vascular leiomyomas that were thought to have been initiated by trauma, although evidence remains anecdotal.^19^

Among the 213 reported cases of leiomyoma in the hand and upper extremity, the majority (approximately 70%) were located on the volar aspect, with only about 10% arising dorsally and the remainder unspecified or deep. In terms of anatomic distribution, 40% involved the digits, 25% the palm or thenar/hypothenar regions, 15% the wrist, and 10% the forearm or arm. This pattern contrasts with that of ganglion cysts, which occur most frequently on the dorsal wrist (60%-70%), followed by the volar wrist (20%) and flexor tendon sheath (10%). The differing distributions reflect their distinct origins: leiomyomas arise from vascular smooth muscle, often adjacent to arteries or nerves, whereas ganglion cysts develop from degenerative changes in the joint capsule or tendon sheath. Nonetheless, clinical overlap may occur at the volar wrist and palmar regions where leiomyomas can present as small, firm, slow-growing masses that closely resemble volar ganglion cysts both clinically and radiographically. Taken together, these findings highlight that leiomyomas exhibit a predominantly volar, vascular, and deep distribution, in contrast to the dorsal, periarticular, and superficial predilection typical of ganglion cysts.

Diagnosing a leiomyoma preoperatively can be challenging due to its nonspecific presentation. Our analysis showed that the misdiagnosis rate was notably high, with 25.2% of patients initially misdiagnosed as having a ganglion cyst. This finding is consistent with the overlap in clinical and radiological features between ganglion cysts and leiomyomas, particularly when they are located in the hand or wrist.^18^ Such misdiagnoses may delay appropriate treatment, emphasizing the importance of considering leiomyomas in the differential diagnosis of soft tissue masses, especially in cases presenting with pain.

Ultrasound and MRI are both crucial for diagnosing angioleiomyomas of the upper extremity, each offering distinct benefits depending on the clinical setting. Ultrasound is rapid, cost-effective, and widely accessible, making it ideal for initial evaluation in resource-limited environments. It is also highly sensitive in the evaluation of soft-tissue masses, including differentiating between benign and malignant lesions, with several studies demonstrating that specific sonographic features such as irregular or infiltrative margins, scalloped contours, and larger size are strongly associated with malignancy.^20-22^ In addition, US allows real-time imaging for guided interventions, such as biopsy, which is essential for histopathological confirmation. For angioleiomyomas, it can identify well-defined, hypoechoic masses with vascular features via Doppler imaging, thus aiding in diagnosis. However, its lower spatial resolution may limit its effectiveness in assessing deeper lesions.

Magnetic resonance imaging, in contrast, provides superior soft tissue contrast and detailed imaging, particularly for large or complex lesions near vital structures. It offers precise information on tumor size, vascularity, and anatomical relationships, which is crucial for surgical planning. However, MRI’s higher cost, longer procedure time, and limited availability make it more suitable for complicated cases where a comprehensive evaluation is required to ensure optimal surgical planning and outcomes.

In resource-limited settings, US should be prioritized for its speed, affordability, and ability to guide further interventions. While MRI remains the gold standard for complex cases, US is an invaluable first-line tool for evaluating soft tissue masses, as demonstrated in our video (Video 1). Cost comparisons between US and MRI are highly variable and context-dependent. The 213 reported cases in this review originated from 26 countries, each with distinct health care systems, insurance structures, access to imaging, and treatment costs. As such, a meaningful cost analysis could not be derived from the available data. In general, US is widely recognized as a more accessible and lower-cost imaging modality compared to MRI, particularly for the initial evaluation of superficial soft-tissue masses. However, no studies were identified that specifically evaluated the cost-effectiveness of imaging approaches or the clinical and financial consequences of misdiagnosis in the context of leiomyomas. This represents an area for future investigation.

Surgical excision remains the gold standard for the treatment of leiomyomas in the upper extremity, as demonstrated by our findings. Historically, a 2- to 3-mm margin of excision was recommended for leiomyomas with vascular involvement.^22^ However, in the reviewed cases, marginal excision with complete enucleation of the tumor and removal from its surrounding pseudocapsule was the most commonly described technique. Given the absence of reported recurrences following marginal excision in the literature, we believe this represents an appropriate and effective treatment approach. The surgical objective should be to carefully separate the lesion from adjacent healthy tissue along the natural cleavage plane between the leiomyoma and the surrounding normal smooth muscle. This finding is consistent with the benign nature of leiomyomas, further reinforcing the efficacy of surgical excision as the preferred treatment modality. The mean follow-up period of 27.3 months in our study indicates that, for most patients, the prognosis after surgery is favorable, with no evidence of recurrence or malignant transformation.

For the majority of the cases reviewed in our study, lesions were typically well-encapsulated and subcutaneous, making them ideal candidates for WALANT surgery (Video 2). The epinephrine used in the local anesthetic of WALANT was sufficient to control bleeding, even in these vascular lesions.

For lesions with perfusion concerns or compression neuropathy symptoms, it is essential to consider the vascularity of these tumors when planning surgical excision. The absence of reported complications in our cohort suggests that, even in challenging cases, careful surgical planning and technique can yield successful outcomes.

This review highlights several noteworthy findings for clinicians managing leiomyomas in the upper extremity. First, the most common presenting symptoms (pain and swelling) are indicative of the compressive and vascular nature of these tumors. The high incidence of misdiagnosis as ganglion cysts underscores the importance of considering leiomyomas in the differential diagnosis of soft tissue masses and utilizing simple diagnostic tools like US. Moreover, although imaging can aid in identifying the lesion’s characteristics, histopathological examination remains the gold standard for diagnosis, reinforcing the need for surgical excision for both diagnostic and therapeutic purposes.

## Conclusion

This systematic review of 213 cases of leiomyomas in the upper extremity provides significant insights into the clinical presentation, diagnostic challenges, and treatment outcomes. Our findings underscore the importance of considering leiomyomas in the differential diagnosis of soft tissue masses, particularly in patients with pain, and reaffirm that surgical excision remains the treatment of choice, offering a favorable prognosis with no reported recurrences in our cohort.

Ultrasound, with its speed, cost-effectiveness, and accessibility, proves especially valuable in resource-limited settings, aiding in rapid differentiation of lesions and guiding interventions like biopsy. Although MRI remains essential for complex cases, US should be prioritized for initial evaluations.

## Treatment Recommendations

1. Ultrasound for diagnosis: in resource-limited settings, US provides a quick and cost-effective first-line diagnostic tool. Doppler assessment can help determine whether the lesion arises from or merely abuts a digital artery or vein, and confirm flow characteristics before surgical planning. When arterial involvement is suspected, MRI or surgical exploration should be considered.
2. Biopsy for diagnosis: excisional biopsy is appropriate for small, superficial, and well-circumscribed lesions typical of leiomyomas. For deeper or atypical lesions where malignancy cannot be excluded, an image-guided core needle biopsy is preferred to minimize morbidity and maintain surgical planes.
3. WALANT surgery: WALANT is ideal for small, superficial, nonadherent lesions that can be excised safely under local anesthesia. Lesions involving or adjacent to critical neurovascular structures, or those requiring extensive dissection or microsurgical reconstruction, may necessitate sedation or general anesthesia based on patient comfort and surgeon preference.
4. Surgical excision: marginal excision with enucleation of the lesion and removal of its pseudocapsule is recommended. No recurrences were reported in the reviewed literature with this technique, suggesting wide margins are unnecessary.
5. Follow-up: although recurrence is rare, clinical follow-up for 12 to 24 months is recommended to confirm healing and monitor for delayed recurrence or functional impairment.

## Supplemental Material

sj-docx-1-han-10.1177_15589447261433068 – Supplemental material for Systematic Review of Leiomyomas of the Upper Extremity: Evaluating the Role of Ultrasound in Preoperative Diagnosis Supplemental material, sj-docx-1-han-10.1177_15589447261433068 for Systematic Review of Leiomyomas of the Upper Extremity: Evaluating the Role of Ultrasound in Preoperative Diagnosis by Kenzie J. MacNeil, Todd W. J. Dow, Katie B. Ross and Donald H. Lalonde in HAND

sj-docx-2-han-10.1177_15589447261433068 – Supplemental material for Systematic Review of Leiomyomas of the Upper Extremity: Evaluating the Role of Ultrasound in Preoperative Diagnosis Supplemental material, sj-docx-2-han-10.1177_15589447261433068 for Systematic Review of Leiomyomas of the Upper Extremity: Evaluating the Role of Ultrasound in Preoperative Diagnosis by Kenzie J. MacNeil, Todd W. J. Dow, Katie B. Ross and Donald H. Lalonde in HAND
