# Supplementary material for: Systematic Review of Leiomyomas of the Upper Extremity: Evaluating the Role of Ultrasound in Preoperative Diagnosis
Source: Hand (N Y). 2026 Apr 7:15589447261433068. Online ahead of print. doi: 10.1177/15589447261433068 (PMC13056795; doi:10.1177/15589447261433068)
Supplement: sj-docx-1-han-10.1177_15589447261433068 – Supplemental material for Systematic Review of Leiomyomas of the Upper Extremity: Evaluating the Role of Ultrasound in Preoperative Diagnosis [file sj-docx-1-han-10.1177_15589447261433068.docx]

| Case Number | Author | Country | Year | Age (Years) | Sex | Side | Location | Duration of Symptoms (Months) | Follow-up Post-Surgery (Months) |
| --- | --- | --- | --- | --- | --- | --- | --- | --- | --- |
| 1 | Mason & Wheelock^1a^ | USA | 1954 | 5 | M | L | Left wrist (volar) | 36 | 84 |
| 2 | Kerr^2a^ | Scotland | 1964 | 32 | F | L | Left forearm (anterolateral surface) | 36 | 60 |
| 3 | Michel & Cretin^3a^ | France | 1964 | 49 | M | R | Right supraclavicular and deltoid region | 300 | N/A |
| 4 | Drew^4a^ | USA | 1966 | 29 | F | R | Right arm (posterior upper arm) | 84 | N/A |
| 5 | Bulmer^5a^ | England | 1967 | 7 | M | R | Right elbow adjacent to radial nerve | 1 | 6 |
| 6 | Ajwani^6a^ | India | 1974 | 50 | M | L | Left hand (hypothenar eminence) | 36 | 60 |
| 7 | Ajwani^6a^ | India | 1974 | 40 | M | L | Left hand (ventral 4th webspace) | 6 | 36 |
| 8 | Hauswald et al.^7a^ | USA | 1975 | 44 | M | R | Right hand (first web space) | 60 | No |
| 9 | Firpo et al.^8a^ | USA | 1976 | 50 | M | L | Left ring finger (volar) - close proximity to collateral vessel | 180 | 48 |
| 10 | Firpo et al.^8a^ | USA | 1976 | 65 | M | L | MCP fold of left index finger (palmer) | 216 | 12 |
| 11 | Neviaser & Newman^9a^ | USA | 1977 | 46 | N/A | N/A | Forearm | N/A | N/A |
| 12 | Neviaser & Newman^9a^ | USA | 1977 | 46 | N/A | N/A | Forearm | N/A | N/A |
| 13 | Neviaser & Newman^9a^ | USA | 1977 | 46 | N/A | N/A | Forearm | N/A | N/A |
| 14 | Neviaser & Newman^9a^ | USA | 1977 | 46 | N/A | N/A | Forearm | N/A | N/A |
| 15 | Neviaser & Newman^9a^ | USA | 1977 | 46 | N/A | N/A | Forearm | N/A | N/A |
| 16 | Neviaser & Newman^9a^ | USA | 1977 | 46 | N/A | N/A | Forearm | N/A | N/A |
| 17 | Neviaser & Newman^9a^ | USA | 1977 | 46 | N/A | N/A | Forearm | N/A | N/A |
| 18 | Neviaser & Newman^9a^ | USA | 1977 | 46 | N/A | N/A | Forearm | N/A | N/A |
| 19 | Neviaser & Newman^9a^ | USA | 1977 | 46 | N/A | N/A | Forearm | N/A | N/A |
| 20 | Neviaser & Newman^9a^ | USA | 1977 | 46 | N/A | N/A | Forearm | N/A | N/A |
| 21 | Neviaser & Newman^9a^ | USA | 1977 | 46 | N/A | N/A | Wrist | N/A | N/A |
| 22 | Neviaser & Newman^9a^ | USA | 1977 | 46 | N/A | N/A | Wrist | N/A | N/A |
| 23 | Neviaser & Newman^9a^ | USA | 1977 | 46 | N/A | N/A | Hand | N/A | N/A |
| 24 | Neviaser & Newman^9a^ | USA | 1977 | 46 | N/A | N/A | Hand | N/A | N/A |
| 25 | Neviaser & Newman^9a^ | USA | 1977 | 46 | N/A | N/A | Hand | N/A | N/A |
| 26 | Neviaser & Newman^9a^ | USA | 1977 | 46 | N/A | N/A | Hand | N/A | N/A |
| 27 | Neviaser & Newman^9a^ | USA | 1977 | 46 | N/A | N/A | Hand | N/A | N/A |
| 28 | Neviaser & Newman^9a^ | USA | 1977 | 46 | N/A | N/A | Hand | N/A | N/A |
| 29 | Neviaser & Newman^9a^ | USA | 1977 | 46 | N/A | N/A | Hand | N/A | N/A |
| 30 | Neviaser & Newman^9a^ | USA | 1977 | 46 | N/A | N/A | Hand | N/A | N/A |
| 31 | Neviaser & Newman^9a^ | USA | 1977 | 46 | N/A | N/A | Hand | N/A | N/A |
| 32 | Neviaser & Newman^9a^ | USA | 1977 | 46 | N/A | N/A | Hand | N/A | N/A |
| 33 | Neviaser & Newman^9a^ | USA | 1977 | 46 | N/A | N/A | Hand | N/A | N/A |
| 34 | Neviaser & Newman^9a^ | USA | 1977 | 46 | N/A | N/A | Hand | N/A | N/A |
| 35 | Cherubino^10a^ | Italy | 1982 | 55 | M | R | Right thenar eminence | 24 | N/A |
| 36 | Vleazco & Marks^11a^ | USA | 1982 | 32 | M | N/A | Dorsum of PIPJ of index finger | 3 | 12 |
| 37 | Duinslaeger et al.^12a^ | Belgium | 1987 | 68 | M | L | Left hand (thenar region) | 204 | 12 |
| 38 | Duinslaeger et al.^12a^ | Belgium | 1987 | 27 | M | R | MCP joint of right index finger | 24 | No |
| 39 | Tyszka et al.^13a^ | Poland | 1987 | 46 | M | R | Right D2 PP volar | 240 | N/A |
| 40 | Sahapiro & Froimson^14a^ | USA | 1988 | 79 | F | R | Right hand (volar third web space) | N/A | N/A |
| 41 | Bottle & Silver^15a^ | USA | 1989 | 62 | M | R | Ulnopalmar side of the right index finger at the level of the PIP. Mass was an enlargement of the ulnar digital artery | 36 | 42 |
| 42 | Freedman & Meland^16a^ | USA | 1989 | 46 | F | R | Right fourth dorsal webspace | 36 | N/A |
| 43 | Marti & Otto^17a^ | Switzerland | 1989 | 38 | M | L | Left D4 DP | N/A | N/A |
| 44 | Duport et al.^18a^ | France | 1990 | 58 | F | L | Left guyon canal - involvement ulnar nerve | N/A | 1 |
| 45 | Tsukagoshi^19a^ | Japan | 1990 | 69 | N/A | L | Left hypothenar | 6 | N/A |
| 46 | Vaughn et al.^20a^ | USA | 1990 | 46 | M | R | Dorsal right ring finger | 84 | N/A |
| 47 | Uchida et al.^21a^ | Japan | 1992 | 14 | F | L | Left arm (posterior) | 6 | 72 |
| 48 | Uchida et al.^21a^ | Japan | 1992 | 44 | M | R | Right little finger (volar) | 2 | 72 |
| 49 | Uchida et al.^21a^ | Japan | 1992 | 50 | M | L | Left palm | 36 | 72 |
| 50 | Uchida et al.^21a^ | Japan | 1992 | 26 | M | L | Left index finger (volar) | 24 | 72 |
| 51 | Uchida et al.^21a^ | Japan | 1992 | 65 | F | R | Right ring finger (volar) | 24 | 72 |
| 52 | Uchida et al.^21a^ | Japan | 1992 | 35 | F | L | Left index finger (dorsal) | 36 | 72 |
| 53 | Uchida et al.^21a^ | Japan | 1992 | 34 | F | L | Left forearm (anterior) | 2 | 72 |
| 54 | Uchida et al.^21a^ | Japan | 1992 | 48 | M | R | Right thumb (volar) | 2 | 72 |
| 55 | Uchida et al.^21a^ | Japan | 1992 | 49 | F | R | Right index finger (dorsal) | 5 | 72 |
| 56 | Uchida et al.^21a^ | Japan | 1992 | 26 | M | R | Right palm | 120 | 72 |
| 57 | Uchida et al.^21a^ | Japan | 1992 | 35 | F | R | Right palm | 2 | 72 |
| 58 | Boyd et al.^22a^ | USA | 1994 | 18 | F | R | Base of right fourth finger (palmar aspect of 4th MCP joint) | 12 | N/A |
| 59 | Calle et al.^23a^ | USA | 1994 | 38 | M | L | Left ring finger (anterolateral aspect of proximal phalanx) | 60 | 24 |
| 60 | Calle et al.^23a^ | USA | 1994 | 36 | M | L | Left index finger (ulnar side of proximal phalanx) | 0.5 | N/A |
| 61 | Calle et al.^23a^ | USA | 1994 | 39 | M | L | Ulnar aspect of left ring finger distal to the proximal interphalangeal joint | 180 | N/A |
| 62 | Calle et al.^23a^ | USA | 1994 | 47 | F | R | Right first webspace superficial to adductor muscle | 4 | N/A |
| 63 | Calle et al.^23a^ | USA | 1994 | 61 | M | L | Hypothenar eminence left hand | 24 | N/A |
| 64 | Glowacki & Weiss^24a^ | USA | 1995 | 73 | M | R | Right volar proximal phalanx of index finger | 36 | 24 |
| 65 | Herren et al.^25a^ | Switzerland | 1995 | 17 | M | L | Pulp of left index finer | 36 | 48 |
| 66 | Lawson et al.^26a^ | UK | 1995 | 37 | M | N/A | Subcutaneous - 2nd dorsal webspace | 24 | N/A |
| 67 | Lawson et al.^26a^ | UK | 1995 | 16 | M | N/A | Subcutaneous - 2nd volar webspace | 2 | N/A |
| 68 | Lawson et al.^26a^ | UK | 1995 | 62 | M | N/A | Subcutaneous - mid dorsal hand | 36 | N/A |
| 69 | Lawson et al.^26a^ | UK | 1995 | 43 | M | N/A | Subcutaneous - just proximal to D2 PIPJ | 24 | N/A |
| 70 | Lawson et al.^26a^ | UK | 1995 | 65 | M | N/A | Subcutaneous - 4th dorsal webspace | 72 | N/A |
| 71 | Lawson et al.^26a^ | UK | 1995 | 52 | M | N/A | Subcutaneous - hypothenar region | 2 | N/A |
| 72 | Lawson et al.^26a^ | UK | 1995 | 34 | F | N/A | Subcutaneous - 1st volar webspace | 144 | N/A |
| 73 | Lawson et al.^26a^ | UK | 1995 | 62 | F | N/A | Subcutaneous - dorsal D1 PIPJ | 18 | N/A |
| 74 | Lawson et al.^26a^ | UK | 1995 | 62 | F | N/A | Dermal - thenar region | 48 | N/A |
| 75 | Lawson et al.^26a^ | UK | 1995 | 86 | F | N/A | Dermal - thenar region | 48 | N/A |
| 76 | Lawson et al.^26a^ | UK | 1995 | 44 | F | N/A | Dermal - volar D3 DIPJ | 12 | N/A |
| 77 | Lawson et al.^26a^ | UK | 1995 | 28 | F | N/A | Dermal - volar D4 PIPJ | 8 | N/A |
| 78 | Lawson et al.^26a^ | UK | 1995 | 70 | M | N/A | Subcutaneous - volar proximal hypothenar region | 72 | N/A |
| 79 | Lawson et al.^26a^ | UK | 1995 | 43 | M | N/A | Dermal - volar D4 MCPJ | 8 | N/A |
| 80 | Piers et al.^27a^ | USA | 1996 | 84 | M | L | Volar left wrist (just proximal to the distal wrist crease on the ulnar side of the palmaris longus tendon) | N/A | 48 |
| 81 | Yang et al.^28a^ | USA | 1996 | 10 | M | R | Center of right palm | N/A | 36 |
| 82 | Garofalo^29a^ | UK | 1997 | 56 | F | L | LEft palm and dorsum hand | 120 | N/A |
| 83 | Kataoka et al.^30a^ | Japan | 1997 | 73 | M | R | Thenar region of right hand | 180 | 36 |
| 84 | Hwang et al.^31a^ | Korea | 1998 | 41 | M | R | Flexor tendon sheath of the right hand | 60 | N/A |
| 85 | Hwang et al.^31a^ | Korea | 1998 | 71 | M | L | Flexor tendon sheath of the left hand | 36 | N/A |
| 86 | Ardito et al.^32a^ | Germany | 1999 | 52 | F | R | Right D4 | 60 | 7 |
| 87 | Jougla et al.^33a^ | France | 1999 | 69 | F | L | Palmar D5 left hand | 120 | N/A |
| 88 | Okamoto et al.^34a^ | Japan | 2000 | 61 | F | R | Dorsal right hand | 24 | N/A |
| 89 | Billings et al.^35a^ | USA | 2001 | 14 | F | N/A | Forearm | N/A | 68 |
| 90 | Gassel et al.^36a^ | Germany | 2001 | 62 | M | L | Left palm CMC joint | 24 | N/A |
| 91 | Scapinelli et al.^37a^ | UK | 2001 | 61 | F | R | Volar aspect of right forearm (radial aspect between mid third and distal third) | 204 | 30 |
| 92 | Scapinelli et al.^37a^ | UK | 2001 | 62 | M | R | Dorsal surface of right hand | "Several years" | 14 |
| 93 | Lim et al.^38a^ | Korea | 2002 | 62 | M | R | Volar surface of right index finger (over proximal phalanx) | 120 | N/A |
| 94 | Dominguez-Cherit & Brandariz^39a^ | Mexico | 2003 | 45 | M | R | Distal phalanx right fourth finger | 12 | 36 |
| 95 | Moritomo et al.^40a^ | Japan | 2003 | 71 | M | R | Right palm (distal aspect) | Since adolescence | N/A |
| 96 | Kugimoto et al.^41a^ | Kugimoto | 2004 | 72 | M | R | Right forearm involving ulnar artery | 120 | 36 |
| 97 | Ramesh et al.^42a^ | UK | 2004 | 39 | M | L | Left hand (ulnar border at base of little finger) | 6 | N/A |
| 98 | Ramesh et al.^42a^ | UK | 2004 | 39 | M | L | Left palm | 12 | 12 |
| 99 | Ramesh et al.^42a^ | UK | 2004 | 30 | M | L | Left palm | 18 | N/A |
| 100 | Yang et al.^43a^ | Taiwan | 2004 | 59 | F | R | Right third finger (distal palmar crease) | N/A | 12 |
| 101 | Zikria et al.^44a^ | USA | 2004 | 38 | F | L | Left wrist (distal aspect of the ulna) | 156 | 26 |
| 102 | Nakamura et al.^45a^ | Japan | 2005 | 74 | F | L | left | 12 | N/A |
| 103 | Oktem^46a^ | Turkey | 2005 | 29 | M | L | Left palm - between 2 & 3 MC | N/A | N/A |
| 104 | Kang et al.^47a^ | Korea | 2006 | 31 | M | L | Left D3 | N/A | 7 |
| 105 | Maresca et al.^48a^ | Italy | 2006 | 12 | M | R | Right D5 volar | 12 | 6 |
| 106 | Nagata et al.^49a^ | Japan | 2006 | 72 | M | L | Left palm | 96 | N/A |
| 107 | Yagi et al.^50a^ | Japan | 2006 | 65 | F | N/A | It arose as an ovoid vascular-rich mass from both the deep palmar arch and the second common palmar digital artery | 24 | 12 |
| 108 | Chalidis & Dimitriou^51a^ | Greece | 2007 | 32 | M | L | Volar surface of left hand and fingers | 24 | 24 |
| 109 | Del Olmo et al.^52a^ | Spain | 2007 | 18 | M | L | Left D3 | 84 | N/A |
| 110 | Boutayeb et al.^53a^ | Morocco | 2008 | 70 | F | R | Right index Finger | N/A | 6 |
| 111 | Huang & Lee^54a^ | Taiwan | 2008 | 11 | M | R | right FDS of D2 | 12 | 12 |
| 112 | Kacerovska et al.^55a^ | Czech | 2008 | 70 | F | L | Left second finger | 12 | N/A |
| 113 | Miyamoto et al.^56a^ | Japan | 2008 | 79 | M | L | Left hypothenar eminence | 24 | 24 |
| 114 | Harb et al.^57a^ | UK | 2009 | 39 | M | R | Right D4 | 3 | 18 |
| 115 | Kulkarni et al.^58a^ | USA | 2009 | 10 | M | R | Volar base right D3 | 12 | 6 |
| 116 | Kulkarni et al.^58a^ | USA | 2009 | 54 | F | L | Volar base left D3 | 12 | N/A |
| 117 | Kulkarni et al.^58a^ | USA | 2009 | 48 | F | R | Right palm base D2 and D3 | 6 | N/A |
| 118 | Park et al.^59a^ | US | 2009 | 42 | M | L | Left thumb | Several months |  |
| 119 | Jeong et al.^60a^ | Korea | 2010 | 50 | M | R | Right Hypothenar eminence, Guyon canal | 3 | 4 |
| 120 | Shafi et al.^61a^ | Japan | 2010 | 37 | M | R | Right hypothenar area | N/A | 12 |
| 121 | Hiromatsu et al.^62a^ | Japan | 2011 | 39 | M | L | Radial aspect of proximal phalanx of left ring finger | 120 | 60 |
| 122 | Miranda et al.^63a^ | Brazil | 2012 | 30 | M | N/A | Palmar aspect of middle phalanx of index finger | 36 | N/a |
| 123 | Houdek et al.^64a^ | USA | 2013 | 55 | M | N/A | hand (n = 14), wrist (1), forearm (n = 5), elbow (n = 4), arm (n = 2) | 72 | 8.6 |
| 124 | Houdek et al.^64a^ | USA | 2013 | 55 | M | N/A | hand (n = 14), wrist (1), forearm (n = 5), elbow (n = 4), arm (n = 2) | 72 | 8.6 |
| 125 | Houdek et al.^64a^ | USA | 2013 | 55 | M | N/A | hand (n = 14), wrist (1), forearm (n = 5), elbow (n = 4), arm (n = 2) | 72 | 8.6 |
| 126 | Houdek et al.^64a^ | USA | 2013 | 55 | M | N/A | hand (n = 14), wrist (1), forearm (n = 5), elbow (n = 4), arm (n = 2) | 72 | 8.6 |
| 127 | Houdek et al.^64a^ | USA | 2013 | 55 | M | N/A | hand (n = 14), wrist (1), forearm (n = 5), elbow (n = 4), arm (n = 2) | 72 | 8.6 |
| 128 | Houdek et al.^64a^ | USA | 2013 | 55 | M | N/A | hand (n = 14), wrist (1), forearm (n = 5), elbow (n = 4), arm (n = 2) | 72 | 8.6 |
| 129 | Houdek et al.^64a^ | USA | 2013 | 55 | M | N/A | hand (n = 14), wrist (1), forearm (n = 5), elbow (n = 4), arm (n = 2) | 72 | 8.6 |
| 130 | Houdek et al.^64a^ | USA | 2013 | 55 | M | N/A | hand (n = 14), wrist (1), forearm (n = 5), elbow (n = 4), arm (n = 2) | 72 | 8.6 |
| 131 | Houdek et al.^64a^ | USA | 2013 | 55 | M | N/A | hand (n = 14), wrist (1), forearm (n = 5), elbow (n = 4), arm (n = 2) | 72 | 8.6 |
| 132 | Houdek et al.^64a^ | USA | 2013 | 55 | M | N/A | hand (n = 14), wrist (1), forearm (n = 5), elbow (n = 4), arm (n = 2) | 72 | 8.6 |
| 133 | Houdek et al.^64a^ | USA | 2013 | 55 | M | N/A | hand (n = 14), wrist (1), forearm (n = 5), elbow (n = 4), arm (n = 2) | 72 | 8.6 |
| 134 | Houdek et al.^64a^ | USA | 2013 | 55 | M | N/A | hand (n = 14), wrist (1), forearm (n = 5), elbow (n = 4), arm (n = 2) | 72 | 8.6 |
| 135 | Houdek et al.^64a^ | USA | 2013 | 55 | F | N/A | hand (n = 14), wrist (1), forearm (n = 5), elbow (n = 4), arm (n = 2) | 72 | 8.6 |
| 136 | Houdek et al.^64a^ | USA | 2013 | 55 | F | N/A | hand (n = 14), wrist (1), forearm (n = 5), elbow (n = 4), arm (n = 2) | 72 | 8.6 |
| 137 | Houdek et al.^64a^ | USA | 2013 | 55 | F | N/A | hand (n = 14), wrist (1), forearm (n = 5), elbow (n = 4), arm (n = 2) | 72 | 8.6 |
| 138 | Houdek et al.^64a^ | USA | 2013 | 55 | F | N/A | hand (n = 14), wrist (1), forearm (n = 5), elbow (n = 4), arm (n = 2) | 72 | 8.6 |
| 139 | Houdek et al.^64a^ | USA | 2013 | 55 | F | N/A | hand (n = 14), wrist (1), forearm (n = 5), elbow (n = 4), arm (n = 2) | 72 | 8.6 |
| 140 | Houdek et al.^64a^ | USA | 2013 | 55 | F | N/A | hand (n = 14), wrist (1), forearm (n = 5), elbow (n = 4), arm (n = 2) | 72 | 8.6 |
| 141 | Houdek et al.^64a^ | USA | 2013 | 55 | F | N/A | hand (n = 14), wrist (1), forearm (n = 5), elbow (n = 4), arm (n = 2) | 72 | 8.6 |
| 142 | Houdek et al.^64a^ | USA | 2013 | 55 | F | N/A | hand (n = 14), wrist (1), forearm (n = 5), elbow (n = 4), arm (n = 2) | 72 | 8.6 |
| 143 | Houdek et al.^64a^ | USA | 2013 | 55 | F | N/A | hand (n = 14), wrist (1), forearm (n = 5), elbow (n = 4), arm (n = 2) | 72 | 8.6 |
| 144 | Houdek et al.^64a^ | USA | 2013 | 55 | F | N/A | hand (n = 14), wrist (1), forearm (n = 5), elbow (n = 4), arm (n = 2) | 72 | 8.6 |
| 145 | Houdek et al.^64a^ | USA | 2013 | 55 | F | N/A | hand (n = 14), wrist (1), forearm (n = 5), elbow (n = 4), arm (n = 2) | 72 | 8.6 |
| 146 | Houdek et al.^64a^ | USA | 2013 | 55 | F | N/A | hand (n = 14), wrist (1), forearm (n = 5), elbow (n = 4), arm (n = 2) | 72 | 8.6 |
| 147 | Houdek et al.^64a^ | USA | 2013 | 55 | F | N/A | hand (n = 14), wrist (1), forearm (n = 5), elbow (n = 4), arm (n = 2) | 72 | 8.6 |
| 148 | Houdek et al.^64a^ | USA | 2013 | 55 | F | N/A | hand (n = 14), wrist (1), forearm (n = 5), elbow (n = 4), arm (n = 2) | 72 | 8.6 |
| 149 | Karaarslan et al.^65a^ | Turkey | 2013 | 22 | M | R | Right FDP of middle finger | N.A | N/A |
| 150 | Kobayashi et al.^66a^ | Japan | 2013 | 57 | M | R | Right superficial palmar arch | 4 | N/A |
| 151 | Nishio et al.^67a^ | Japan | 2013 | 56 | M | R | First web space of right hand | 36 | 4 |
| 152 | Kanthan et al.^68a^ | Canada | 2014 | 71 | M | N/A | Palm | N/A | N/A |
| 153 | Moncef et al.^69a^ | Morocco | 2014 | 30 | M | L | Swelling of the posterior surface radial border of left forearm | N/A | 12 |
| 154 | Ohtsuka^70a^ | Japan | 2014 | 64 | M | R | Pulp of right middle finger | 120 | N/A |
| 155 | Pavard et al.^71a^ | Belgium | 2014 | 35 | M | N/A | Elbow | N/A | N/A |
| 156 | Prasad et al.^72a^ | India | 2014 | 70 | F | N/A | Terminal phalanx of D3 | 180 | N/A |
| 157 | Ramachandran et al.^73a^ | India | 2014 | 16 | M | L | Left forearm | 2 | N/A |
| 158 | Sayit et al.^74a^ | Turkey | 2014 | 58 | M | R | Volar rigth index finger | N/A | 4 |
| 159 | Baker et al.^75a^ | UK | 2015 | 42 | M | R | Volar Middle phalanx right D2 | 18 | N/A |
| 160 | Jing & Giesen^76a^ | UK | 2015 | 63 | F | L | Palmar left wrist within the median nerve | 24 | 12 |
| 161 | Mok et al.^77a^ | USA | 2015 | 77 | F | L | Left middle finger | 240 | 19 |
| 162 | Paluck et al.^78a^ | USA | 2015 | 30 | F | R | Right volar wrist | 2 | 0.25 |
| 163 | Pozzatti et al.^79a^ | Brazil | 2015 | 61 | F | L | Left thumb | 12 | 12 |
| 164 | Bommireddy & Gurram^80a^ | India | 2016 | 25 | F | R | Right forearm | 6 | 24 |
| 165 | Komforti et al.^81a^ | USA | 2016 | 16 | F | L | Left subungal thumb | 2.5 | 18 |
| 166 | Hammond et al.^82a^ | USA | 2017 | 40 | F | N/A | Finger, unspecified | N/A | 44 |
| 167 | Hammond et al.^82a^ | USA | 2017 | N/A | N/A | N/A | Dorsal hand | N/A | 44 |
| 168 | Hammond et al.^82a^ | USA | 2017 | 67 | M | N/A | Dorsum of finger | N/A | 44 |
| 169 | Hammond et al.^82a^ | USA | 2017 | N/A | N/A | N/A | Dorsal Hand | N/A | 44 |
| 170 | Hammond et al.^82a^ | USA | 2017 | 69 | F | N/A | Finger, unspecified | N/A | 44 |
| 171 | Hammond et al.^82a^ | USA | 2017 | 76 | F | N/A | Finger, unspecified | N/A | 44 |
| 172 | Kulkarni et al.^83a^ | India | 2017 | 49 | M | R | Right dorsal hand, second web space | 60 | 24 |
| 173 | Kanta et al.^84a^ | Czech Republic | 2018 | 55 | F | N/A | First indigital space of second and third metacarpal bone within median nerve | 420 | N/A |
| 174 | Oliver et al.^85a^ | USA | 2018 | 44 | F | R | Right thumb base (thenar eminence) | 24 | N/A |
| 175 | Kang et al.^86a^ | Korea | 2019 | N/A | N/A | N/A | Hand and Fingers | N/A | N/A |
| 176 | Kang et al.^86a^ | Korea | 2019 | N/A | N/A | N/A | Hand and Fingers | N/A | N/A |
| 177 | Kang et al.^86a^ | Korea | 2019 | N/A | N/A | N/A | Hand and Fingers | N/A | N/A |
| 178 | Kang et al.^86a^ | Korea | 2019 | N/A | N/A | N/A | Hand and Fingers | N/A | N/A |
| 179 | Kang et al.^86a^ | Korea | 2019 | N/A | N/A | N/A | Wrist | N/A | N/A |
| 180 | Kang et al.^86a^ | Korea | 2019 | N/A | N/A | N/A | Wrist | N/A | N/A |
| 181 | Kang et al.^86a^ | Korea | 2019 | N/A | N/A | N/A | upper arm | N/A | N/A |
| 182 | Kang et al.^86a^ | Korea | 2019 | N/A | N/A | N/A | elbow | N/A | N/A |
| 183 | Kang et al.^86a^ | Korea | 2019 | N/A | N/A | N/A | forewarm | N/A | N/A |
| 184 | Aydın & Berköz^87a^ | Turkey | 2019 | 56 | M | L | Left forearm, from median nerve | N/A | 12 |
| 185 | Lipner & Husain^88a^ | USA | 2019 | 59 | F | L | Left D2 subungal | N/A | 2 |
| 186 | Yeung et al.^89a^ | USA | 2019 | 48.6 | M | N/A | D3 | N/A | 75.8 |
| 187 | Yeung et al.^89a^ | USA | 2019 | 48.6 | M | N/A | D4 | N/A | 75.8 |
| 188 | Yeung et al.^89a^ | USA | 2019 | 48.6 | M | N/A | Fourth websapce | N/A | 75.8 |
| 189 | Yeung et al.^89a^ | USA | 2019 | 48.6 | M | N/A | D2 | N/A | 75.8 |
| 190 | Yeung et al.^89a^ | USA | 2019 | 48.6 | M | N/A | First webspace | N/A | 75.8 |
| 191 | Hernandez et al.^90a^ | Australia | 2020 | 6 | F | R | Right adjacent to FCR | N/A | 6.5 |
| 192 | Jin & Lu^91a^ | China | 2020 | 49 | F | N/A | Finger adjacent to digital nerve | 6 | 37 |
| 193 | Jin & Lu^91a^ | China | 2020 | 56 | M | N/A | Palm adjacent to common digital nerve | 12 | 37 |
| 194 | Jin & Lu^91a^ | China | 2020 | 54 | M | N/A | Palm adjacent to common digital nerve | 120 | 37 |
| 195 | Jin & Lu^91a^ | China | 2020 | 54 | M | N/A | Palm adjacent to common digital nerve | 24 | 37 |
| 196 | Karaman et al.^92a^ | Turkey | 2020 | 16 | M | R | Right FDP D4 | 24 | 12 |
| 197 | Kattan et al.^93a^ | Saudi Arabia | 2020 | 60 | F | R | Right D2 Flexor tendon | N/A | 6 |
| 198 | Lee et al.^94a^ | Korea | 2020 | 37 | M | R | Right upper arm (within radial nerve) | 6 | 12 |
| 199 | Perera et al.^95a^ | Sri Lanka | 2020 | 7 | M | R | Right distal forearm and wrist. The lesion was found to be deep in the pronator quadratus with the muscle adhering to the lesion as a thin sheath | 48 | 6 |
| 200 | Taj et al.^96a^ | USA | 2020 | 68 | M | L | DP Left D3 | 24 | N/A |
| 201 | Brudnik et al.^97a^ | USA | 2021 | 69 | M | R | Right hand dorsum | N/A | N/A |
| 202 | Motiwala & Jivan^98a^ | UKA | 2021 | 40 | M | R | Right D3 | N/A | N/A |
| 203 | Thomas et al.^99a^ | USA | 2021 | 68 | F | L | Left 2nd finger, palmar middle phalanx | 84 | 0.5 |
| 204 | Thomas et al.^99a^ | USA | 2021 | 66 | F | R | Right 5th finger, distal to A1 pulley | N/A | N/A |
| 205 | Thomas et al.^99a^ | USA | 2021 | 60 | F | L | Left 3rd finger, volar ulnar pulp over distal phalanx | 8 | N/A |
| 206 | Lloyd et al.^100a^ | USA | 2022 | 62 | M | L | Left Distal transverse carpal ligament | 180 | 0.5 |
| 207 | AlZahrani et al.^101a^ | Saudi Arabia | 2023 | 69 | F | L | Volar aspect left D1 | 12 | 8 |
| 208 | Amrutiya et al.^102a^ | UK | 2023 | 54 | M | L | Volar left D4 and D5 | 12 | 10 |
| 209 | Ortiz et al.^103a^ | USA | 2023 | 41 | F | L | Left thumb mas | 24 | N/A |
| 210 | Ortiz et al.^103a^ | USA | 2023 | 63 | M | N/A | Radial wrist | N/A | N/A |
| 211 | Warburton et al.^104a^ | USA | 2023 | 52 | F | L | Left wrist (volar) | 24 | N/A |
| 212 | Mercken et al.^105a^ | Belgium | 2024 | 45 | M | N/A | Between third and fourth rays palm | N/A | N/A |
| 213 | MacNeil et al. | Canada | 2025 |  |  |  |  |  |  |

Supplementary Table 1: Reported Cases of Leiomyoma to the Upper Extremity
