# Supplementary material for: Systematic Review of Leiomyomas of the Upper Extremity: Evaluating the Role of Ultrasound in Preoperative Diagnosis
Source: Hand (N Y). 2026 Apr 7:15589447261433068. Online ahead of print. doi: 10.1177/15589447261433068 (PMC13056795; doi:10.1177/15589447261433068)
Supplement: sj-docx-2-han-10.1177_15589447261433068 – Supplemental material for Systematic Review of Leiomyomas of the Upper Extremity: Evaluating the Role of Ultrasound in Preoperative Diagnosis [file sj-docx-2-han-10.1177_15589447261433068.docx]

**References for Reviewed Articles**

**1a**. Mason ML, Wheelock MC. Leiomyoma of flexor tendons of the hand. Quarterly Bulletin of the Northwestern University Medical School. 1954;28(2):124.

**2a**. Kerr IF. Leiomyoma of the basilic vein. Journal of British Surgery. 1964 Nov;51(11):872-3.

**3a**. Michel PJ, Cretin J. Hyperalgic Multiple leiomyomas of the Right shoulder girdle and right arm, with radicular topography. Lyon Medical. 1964 Dec;212:1519-20.

**4a**. Drew EJ. Large leiomyoma of the upper extremity. The American Journal of Surgery. 1966 Dec;112(6):938-40.

**5a**. Bulmer JH. Smooth muscle tumours of the limbs. The Journal of Bone & Joint Surgery British Volume. 1967 Feb;49(1):52-8.

**6a**. Ajwani KD. Angio leiomyoma of the hand: (a report of two cases). Medicine and Surgery. 1974;14(11):14–6.

**7a**. Hauswald KR, Kasdan ML, Weiss DL. Vascular leiomyoma of the hand: case report. Plastic and Reconstructive Surgery. 1975 Jan;55(1):89-91.

**8a**. Firpo CA, Rimoldi MA, Bertole A. Leiomyomas of the hand. International Surgery. 1976 Jan;61(1):45-6.

**9a**. Neviaser RJ, Newman W. Dermal angiomyoma of the upper extremity. The Journal of hand surgery. 1977 Jul;2(4):271-4.

**10a**. Cherubino P. A rare instance of angioleiomyoma of the thenar eminence. Minerva Ortopedica. 1982;33(10):1007–10.

**11a**. Velazco A, Marks TW. Angioleiomyoma of the hand: case report and review of the literature. Orthopedics. 1982 May;5(5):555-7.

**12a**. Duinslaeger L, Vierendeels T, Wylock P. Vascular leiomyoma in the hand. Journal of Hand Surgery. 1987 Jul;12(4):624-7.

**13a**. Tyszka JJ, Stypułkowski TA, Bieniawska M. Leiomyoma diagnosed and treated as a ganglion. Chirurgia Narzadow Ruchu i Ortopedia Polska. 1987 Jan;52(5):402-4.

**14a**. Shapiro JD, Froimson AI. Angiomyoma in the hand. Orthopedics. 1988 Dec;11(12):1709-10.

**15a**. Botte MJ, Silver MA. Leiomyoma of a digital artery. Clinical Orthopaedics and Related Research (1976-2007). 1990 Nov;260:259-62.

**16a**. Freedman AM, Meland NB. Angioleiomyomas of the extremities: report of a case and review of the Mayo Clinic experience. Plastic and reconstructive surgery. 1989 Feb;83(2):328-31.

**17a**. Marti WR, Otto RC. Fibroleiomyoma in a phalanx of the left ring finger. RoeFo-Fortschritte auf dem Gebiete der Roentgenstrahlen und der neuen bildgebenden Verfahren. 1989;151(2):231-2.

**18a**. Duport G, Gayet L, Rovira N, Pries P, Levillain P. Angioleiomyoma developed from the ulnar artery at the wrist. Semaine Des Hopitaux. 1990 Sep;66(34):1955-7.

**19a.** Tsukagoshi T. An Angioleiomyoma of the palm. Japanese Journal of Plastic and Reconstructive Surgery. 1990;33(2):185–8.

**20a**. Vaughn TR, Louton RB, Terranova WT. A large leiomyoma of the digit. Plastic and Reconstructive Surgery. 1990 Sep;86(3):605.

**21a**. Uchida M, Kojima T, Hirase Y, Lizuka T. Clinical characteristics of vascular leiomyoma of the upper extremity: report of 11 cases. British Journal of Plastic Surgery. 1992 Jan;45(7):547-9.

**22a**. Boyd R, Bhatt B, Mandell G, Saxe A. Leiomyoma of the hand: a case report and review of the literature. The Journal of Hand Surgery. 1995 Jan;20(1):24-6.

**23a**. Callé SC, Eaton RG, Littler JW. Vascular leiomyomas in the hand. The Journal of Hand Surgery. 1994 Mar;19(2):281-6.

**24a.** Glowacki KA, Weiss AP. Vascular leiomyoma of the finger causing bone erosion. The Journal of Hand Surgery. 1995 Nov;20(6):1011-3.

**25a**. Herren DB, Zimmermann A, Büchler U. Vascular leiomyoma in an index finger undergoing malignant transformation. Journal of Hand Surgery. 1995 Aug;20(4):484-7.

**26a**. Lawson GM, Salter DM, Hooper G. Angioleiomyomas of the hand: a report of 14 cases. Journal of Hand Surgery. 1995 Aug;20(4):479-83.

**27a**. Piers W, Terrono AL, Hayek J, Millender LH. Angiomyoma (vascular leiomyoma) of the median nerve. Journal of Hand Surgery. 1996;2(21):285-6.

**28a**. Yang SS, Williams RJ, Bear BJ, McCormack RR. Leiomyoma of the Hand in a Child Who Has the Human Immunodeficiency Virus. A Case Report. Journal of Bone and Joint Surgery. 1996 Dec;78(12):1904-6.

**29a**. Garofalo R. A case of Leiomyoma of the left hand. Rivista Italiana di Chirurgia Plastica. 1997;29(3):231–4.

**30a**. Kataoka M, Yano H, Fukunaga T, Masumi S. Giant vascular leiomyoma in the hand. Scandinavian Journal of Plastic and Reconstructive Surgery and Hand Surgery. 1997 Jan 1;31(1):91-3.

**31a**. Hwang JW, Ahn JM, Kang HS, Suh JS, Kim SM, Seo JW. Vascular leiomyoma of an extremity: MR imaging-pathology correlation. American Journal of Roentgenology. 1998 Oct;171(4):981-5.

**32a**. Ardito S, Puzzo L, Giuffrida N. Angiomyoma of the hand. A case report. Minerva Ortopedica e Traumatologica. 1999 Apr;50:59-62.

**33a**. Jougla E, Grolleau JL, Chavoin JP. Angiomyoma of the hand: a post-traumatic tumor? Chirurgie de la Main. 1999;18(3):216‑9.

**34a**. Okamoto Y, Yoshioka H, Takahashi N, Itai Y, Watanabe T. A case of angioleiomyoma in the hand. Japanese Journal of Clinical Radiology. 2000;45(7):897-900.

**35a**. Billings SD, Folpe AL, Weiss SW. Do leiomyomas of deep soft tissue exist? American Journal of Surgical Pathology. 2001;25(9):1134–1142.

**36a**. Gassel F, Kraft CN, Wallny T, Hess L, Schmitt O. Soft‐tissue angioleiomyoma of the hand as a rare differential diagnosis of haemophilic pseudotumour. Haemophilia. 2001 Sep;7(5):528-31.

**37a**. Scapinelli R, Iacobellis C, Taglialavoro G, Blandamura S, Baggio ME. Vascular leiomyoma of the limbs. La Chirurgia Degli Organi di Movimento. 2001 Apr;86(2):143-52.

**38a**. Lim ST, Kim MW, Sohn MH. Tc-99m RBC perfusion and blood-pool scintigraphy in the evaluation of vascular leiomyoma of the hand. Annals of Nuclear Medicine. 2002; 16:293–296.

**39a**. Dominguez‐Cherit J, Brandariz A. Distal digital angioleiomyoma: a case report and review of the literature. International Journal of Dermatology. 2003 Feb;42(2):141-3.

**40a**. Moritomo H, Murase T, Ebara R, Yoshikawa H. Massive vascular leiomyoma of the hand. Scandinavian Journal of Plastic and Reconstructive Surgery and Hand Surgery. 2003 Jan;37(2):125-7.

**41a**. Kugimoto Y, Asami A, Shigematsu M, Hotokebuchi T. Giant vascular leiomyoma with extensive calcification in the forearm. Journal of Orthopaedic Science. 2004 May;9:310-3.

**42a**. Ramesh P, Annapureddy SR, Khan F, Sutaria PD. Angioleiomyoma: a clinical, pathological and radiological review. International Journal of Clinical Practice. 2004 Jun;58(6):587-91.

**43a**. Yang WE, Hsueh S, Chen CH, Lee ZL, Chen WJ. Leiomyoma of the hand mimicking a pearl ganglion. Chang Gung Medical Journal. 2004 Feb;27(2):134-7.

**44a**. Zikria BA, Radevic MR, Jormark SC, Huvos AG, Yang SS. Intraosseous leiomyoma of the ulna: a case report. Journal of Bone and Joint Surgery. 2004 Nov;86(11):2522-5.

**45a**. Nakamura M, Umebayashi Y, Uchihira T, Kamei T, Uchihira N, Muramoto G. Two Case of Angioleiomyoma. Nishi Nihon Hifuka. 2005;67(6):590–3.

**46a**. Oktem F. Vascular leiomyoma of the hand. Plastic and Reconstructive Surgery. 2005 Apr;115(4):1218-9.

**47a**. Kang BS, Kim DH, Cho SH, Lee JD. A Case of digital angioleiomyoma. Korean Journal of Dermatology. 2006:454-6.

**48a**. Maresca A, Gagliano C, Marcuzzi A. Leiomyoma of the hand: a case report. Chirurgie de la Main. 2005 Jun;24(3-4):193-5.

**49a**. Nagata S, Nishimura H, Uchida M, Hayabuchi N, Zenmyou M, Fukahori S. Giant angioleiomyoma in extremity: report of two cases. Magnetic Resonance in Medical Sciences. 2006;5(2):113-8.

**50a**. Yagi K, Hamada Y, Yasui N. A leiomyoma arising from the deep palmar arterial arch. Journal of Hand Surgery. 2006 Dec;31(6):680-2.

**51a**. Chalidis BE, Dimitriou CG. Carpal tunnel syndrome due to an atypical deep soft tissue leiomyoma: The risk of misdiagnosis and mismanagement. World Journal of Surgical Oncology. 2007 Dec;5:1-4.

**52a**. Del Olmo J, Marquina M, Redondo P. Asymptomatic digital angioleiomyoma. Actas Dermosifiliograficas. 2007 May;98(4):292.

**53a**. Boutayeb F, Ibrahimi AE, Chraibi F, Znati K. Leiomyoma in an index finger: report of case and review of literature. Hand. 2008 Sep;3(3):210-1.

**54a**. Huang KC, Lee KF. Angioleiomyoma in the palm of an 11-year-old boy. Skeletal Radiology. 2008 Apr;37:339-41.

**55a**. Kacerovska D, Michal M, Kreuzberg B, Mukensnabl P, Kazakov D. Acral calcified vascular leiomyoma of the skin: a rare clinicopathological variant of cutaneous vascular leiomyomas: report of 3 cases. Journal of the American Academy of Dermatology. 2008;59:1000–1004.

**56a**. Miyamoto W, Yamamoto S, Kii R, Uchio Y. Vascular leiomyoma resulting in ulnar neuropathy: case report. The Journal of Hand Surgery. 2008 Dec;33(10):1868-70.

**57a**. Harb Z, Bismil Q, Ricketts DM. Trigger finger presenting secondary to leiomyoma: a case report. Journal of Medical Case Reports. 2009 Dec;3:1-3.

**58a**. Kulkarni AR, Haase SC, Chung KC. Leiomyoma of the hand. Hand. 2009 Jun;4(2):145-9.

**59a**. Park IJ, Kim HM, Lee HJ. Re: Angioleiomyoma in the digit causing bony destruction. Journal of Hand Surgery (European Volume). 2009 Feb;34(1):131-2.

**60a.** Jeong C, Kim HM, Park IJ. Compression of the ulnar nerve in Guyon’s canal by an angioleiomyoma. Journal of Hand Surgery. 2010 Sep 1;35(7).594-5.

**61a**. Shafi M, Hattori Y, Doi K. Angioleiomyoma of distal ulnar artery of the hand. Hand. 2010 Mar;5(1):82-5.

**62a**. Hiromatsu S, Nata S, Tobinaga S, Aoyagi S. A case of angioleiomyoma presenting as a pulsatile tumor in the left ring finger. Annals of Vascular Surgery. 2011 Jul;25(5):698-e9.

**63a**. Miranda LQ, Rehfeldt FVS, Silva RS, Fonseca JCM, Alves MFGS. Case for diagnosis. Anais Brasileiros de Dermatologia. 2012;87:322–3.

**64a.** Houdek MT, Rose PS, Shon W, Kakar S. Angioleiomyoma of the upper extremity. The Journal of Hand Surgery. 2013 Aug 1;38(8):1579-83.

**65a.** Karaarslan AA, Öztürk AM, Sesli E. Triggering of the finger at the wrist due to a leiomyoma arising from the lumbrical muscle of the middle finger. Journal of Hand Surgery (European Volume). 2014 Sep;39(7):778-9.

**66a**. Kobayashi K, Otsuka H, Fukasawa K. Angioleiomyoma of the superficial palmar arterial arch. Hand Surgery. 2013;18(01):121-3.

**67a**. Nishio J, Aoki M, Tanaka Y, Iwasaki H, Naito M. Painless angioleiomyoma of the first web space of the hand. In Vivo. 2013 Jul;27(4):519-22.

**68a.** Kanthan R, Senger JL, Classen D. Unusual palmar lesions-Myopericytoma, Myofibroma, Angioleiomyoma: Surgical management of a pathological conundrum. Virchows Archiv. 2014 Aug;465:53-54.

**69a.** Moncef E, Abdessamad K, Najib A, Yacoubi H. Vascular leiomyoma of the forearm: presentation of a case report and review of literature. The Pan African Medical Journal. 2014 Oct;19:222-4.

**70a.** Ohtsuka H. Angioleiomyoma of the pulp. Journal of Plastic Surgery and Hand Surgery. 2014 Aug 1;48(4):285-6.

**71a.** Pavard X, Dallaudière B, Omoumi P, Lecouvet FE, Cyteval C, Larbi A. Angioleiomyoma of the elbow. Journal of the Belgian Society of Radiology. 2014 Mar;97(2):124-6.

**72a**. Prasad R, Bhamidi A, Kumar RA, Muthukumar S. Angioleiomyoma of middle finger terminal phalanx—Case report and review of literature. Journal of Hand and Microsurgery. 2014 Jun;6(01):45-6.

**73a.** Ramachandran R, Rangaswami R, Raja DK, Shanmugasundaram G. Deep soft-tissue leiomyoma of the forearm mimicking a primary bone tumor of the ulna. Radiology Case Reports. 2014 Jan;9(3):960.

**74a.** Sayit E, Sayit AT, Zan E, Bakirtas M, Akpinar H, Gunbey P. Vascular leiomyoma of an extremity: Report of two cases with MRI and histopathologic correlation. Journal of Clinical Orthopedic Trauma. 2014;5:110–4.

**75a.** Baker BG, Wain RA, Kanitkar S. Angioleiomyoma arising from a digital artery with demonstrable intra-operative anatomy. European Journal of Plastic Surgery. 2015 Dec;38:507-10.

**76a.** Jing SS, Giesen T. Intraneural angioleiomyoma of the median nerve at the wrist. Journal of Hand Surgery (European Volume). 2015 Jul;40(6):639-40.

**77a.** Mok JC, Fan KY, Lui TH. A huge angioleiomyoma of the finger. Hand Surgery. 2015 Jun;20(02):310-2.

**78a.** Paluck M, Hager N, Gellhorn AC. Sonographic evaluation of trigger finger at the wrist and carpal tunnel syndrome resulting from a deep soft tissue leiomyoma. Journal of Ultrasound in Medicine. 2015 Mar;34(3):545-7.

**79a.** Pozzatti RR, Cordeiro CP, da Cruz JD, de Araújo GC. Leiomyoma in the thumb causing trigger finger. Case Reports. 2015 Aug 3;2015:bcr2015209449.

**80a.** Bommireddy B, Gurram V. Deep soft tissue leiomyoma of forearm: A case report and review of literature. Journal of Clinical and Diagnostic Research. 2016 Jun;10(6):RD03.

**81a.** Komforti M, Selim MA, Bellet JS. Subungual leiomyoma in the left thumb of a 16‐year‐old female. Journal of Cutaneous Pathology. 2016 Apr;43(4):379-82.

**82a.** Hammond MI, Miner AG, Piliang MP. Acral and digital angioleiomyomata: 14‐year experience at the Cleveland Clinic and review of the literature. Journal of Cutaneous Pathology. 2017 Apr;44(4):342-5.

**83a.** Kulkarni MS, Vijayan S, Naik M, Rao SK. A rare tumour of hand: angioleiomyoma. Case Reports. 2017 Apr 28;2017:bcr-2017.

**84a.** Kanta M, Ehler E, Vališ M, Kašparová P, Adamkov J, Klímová B. Leiomyoma of the palm. Ceska A Slovenska Neurologie A Neurochirurgie. 2018 Jan;81(1):93-4.

**85a**. Oliver JD, Boczar D, Huayllani MT, Restrepo DJ, Sisti A, Manrique OJ, Broer PN, Forte AJ. Primary hand leiomyoma: a systematic review. Annals of Plastic Surgery. 2019 Dec 1;83(6):e77-84.

**86a.** Kang BS, Shim HS, Kim JH, Kim YM, Bang M, Lim S, Park GM, Lee TY, Ha ND, Kwon WJ. Angioleiomyoma of the extremities: Findings on ultrasonography and magnetic resonance imaging. Journal of Ultrasound in Medicine. 2019 May;38(5):1201-8.

**87a.** Aydın HU, Berköz Ö. Intraneural angioleiomyoma of the median nerve presenting as a forearm mass: A case report. Acta Orthopaedica et Traumatologica Turcica. 2019 Jul;53(4):310-2.

**88a**. Lipner SR, Husain S. Subungual leiyomyoma presenting as erythronychia: Case report and review of the literature. Journal of Drugs in Dermatology. 2019 May;18(5):465-7.

**89a.** Yeung CM, Moore L, Lans J, Lozano-Calderón L. Angioleiomyoma of the hand: A case series and review of the literature. Archives of Bone and Joint Surgery. 2020 May;8(3):373.

**90a.** Hernandez K, East B, Hutchinson M, Smith M, Williams N. Unique case of 'trigger wrist secondary to deep tissue leiomyoma in a healthy 6‐year‐old. ANZ Journal of Surgery. 2020 Jan;90:176-8.

**91a.** Jin Q, Lu H. Angioleiomyoma of the hand with nerve compression. Journal of International Medical Research. 2020 Jun;48(6):0300060520928683.

**92a.** Karaman İ, Kafadar İ, Günay AE. Leiomyoma of the hand in an adolescent. Journal of Clinical Practice and Research. 2020;42(1):105.

**93a.** Kattan AE, Arab K, Alswayyed MA, Algadiem E, Mardan QN. Angioleiomyoma of the proper ulnar digital artery: Case report. International Journal of Surgery Case Reports. 2020 Jan;71:41-4.

**94a**. Lee BC, Kim HJ, La Choi Y, Jeon BJ, Sung DH. Radial neuropathy caused by intraneural leiomyoma: A case report. Medicine. 2020 May;99(22):e20196.

**95a.** Perera NN, Wijayasinghe SR, Dissanayake K, Fernando J, De Silva ET. Calcified leiomyoma of the distal forearm in a child: A case report and review of literature. Case Reports in Orthopedics. 2020 Aug;2020(1):8821265.

**96a.** Taj H, Comba I, Vasquez J, Zayat V. A rare case of angioleiomyoma of the hand. Cureus. 2020 Apr;12(4).

**97a.** Brudnik R, Shurbaji MS, Vellila RE. Cutaneous angiomyolipoma (Angiolipoleiomyoma): Case report and review of literature with emphasis on nomenclature. American Journal Of Clinical Pathology. 2021 Oct;156:S45.

**98a**. Motiwala F, Jivan S. Digital angioleiomyoma: A rare tumour of the hand. British Journal of Surgery. 2021 Sept;108:259-348.

**99a.** Thomas KD, Ro JY, Ayala AG. Leiomyoma of digit of hand: Report of three cases with literature review. Annals of Diagnostic Pathology. 2021 Feb;50:151669.

**100a.** Lloyd AR, Lohse G, Pourcho AM. The role of point‐of‐care sonographic evaluation in the identification and management of rare angioleiomyoma of the hand: A case report. PM&R: Journal of Injury, Function & Rehabilitation. 2022 Jan;14(1).

**101a.** AlZahrani AM, AlMarshad FA, Fayi K, Alsaud NN, Alkhater JN, Awad MR, Jarman A. Leiomyoma of the thumb mimicking a ganglion: A case report. Cureus. 2023 May;15(5).

**102a.** Amrutiya PA, Brown OS, Papanikos E, Mallina R. Angioleiomyoma–A case report. The Journal of Hand Surgery (Asian-Pacific Volume). 2023 Oct;28(05):614-8.

**103a.** Ortiz WJ, Eager JJ, Cervantes M. Angioleiomyoma of the hand: A report of two cases. Cureus. 2023 Mar;15(3).

**104a.** Warburton C, Patel N, Harris G, Gabor N, Rosenberg AE, Dodds SD, Jose J. Angioleiomyoma in a 52-year-old female wrist: A case report. Radiology Case Reports. 2023 Aug 1;18(8):2663-6.

**105a.** Mercken K, Deschuyffeleer S, Matthys P. A rare case of angioleiomyoma of the palm. Journal of the Belgian Society of Radiology. 2024 May 3;108(1):47.
